# The Impact of Hydrogenation on Structural and Superconducting Properties of FeTe_0.65_Se_0.35_ Single Crystals

**DOI:** 10.3390/ma14247900

**Published:** 2021-12-20

**Authors:** Stanislav I. Bondarenko, Anatolij I. Prokhvatilov, Roman Puźniak, Jarosław Piętosa, Andrey A. Prokhorov, Vladimir V. Meleshko, Valeriy P. Timofeev, Valentin P. Koverya, Dariusz Jakub Gawryluk, Andrzej Wiśniewski

**Affiliations:** 1 B. Verkin Institute for Low Temperature Physics and Engineering, National Academy of Sciences of Ukraine, Nauky 47, 61103 Kharkov, Ukraine; bondarenko@ilt.kharkov.ua (S.I.B.); prokhvatilov@ilt.kharkov.ua (A.I.P.); meleshko@ilt.kharkov.ua (V.V.M.); timofeev@ilt.kharkov.ua (V.P.T.); koverya@ilt.kharkov.ua (V.P.K.); 2 Institute of Physics, Polish Academy of Sciences, Al. Lotników 32/46, 02-668 Warsaw, Poland; pietosa@ifpan.edu.pl (J.P.); gawryluk@ifpan.edu.pl (D.J.G.); wisni@ifpan.edu.pl (A.W.); 3 Institute of Physics of the Czech Academy of Sciences, Na Slovance, 182 21 Prague 8, Czech Republic; andaprokhorov@gmail.com; 4 Laboratory for Multiscale Materials Experiments, Paul Scherrer Institute, 5232 Villigen, Switzerland

**Keywords:** iron-based superconductors, hydrogenation, critical currents, structural properties

## Abstract

Properties of FeTe_0.65_Se_0.35_ single crystals, with the onset of critical temperature (*T*_c_^onset^) at 15.5 K, were modified via hydrogenation performed for 10–90 h, at temperatures ranging from 20 to 250 °C. It was found that the tetragonal matrix became unstable and crystal symmetry lowered for the samples hydrogenated already at 200 °C. However, matrix symmetry was not changed and the crystal was not destroyed after hydrogenation at 250 °C. Bulk *T*_c_^bulk^, determined at the middle of the superconducting transition, which is equal to 12–13 K for the as grown FeTe_0.65_Se_0.35_, rose by more than 1 K after hydrogenation. The critical current density studied in magnetic field up to 70 kOe increased 4–30 times as a consequence of hydrogenation at 200 °C for 10 h. Electron paramagnetic resonance measurements also showed higher values of *T*_c_^bulk^ for hydrogenated crystals. Thermal diffusion of hydrogen into the crystals causes significant structural changes, leads to degeneration of crystal quality, and significantly alters superconducting properties. After hydrogenation, a strong correlation was noticed between the structural changes and changes in the parameters characterizing the superconducting state.

## 1. Introduction

Nowadays, hydrogen-reach superconducting compounds constitute a very active research area. Over past few years, high values of superconducting transition temperature, close to room temperature, have been found mainly in those compounds subjected to high external pressure [1,2,3,4,5]. On the other hand, at ambient pressure, for many compounds, e.g., metallic, carbon-based, cuprate, pnictide materials, hydrogenation leads to a significant improvement of their superconducting properties [6]. Particularly in BCS superconductors, hydrogen introduces new phonon modes, which strongly affect superconducting properties due to anharmonicity of hydrogen-related vibrations. Additionally, hydrogen is a very useful tool in testing mechanisms of superconductivity in terms of isotope effect and neutron scattering studies—available deuterium isotope has doubled atomic mass and a greater scattering cross-section, respectively.

It was found that after hydrogenation, flux pinning properties of superconductors improve. For example, it was shown that lattice defects, including dislocations, grain boundaries, and impurities such as hydrogen that generate hydride precipitates, effectively pin the vortices inside the material [7,8]. The effect of hydrogenation on properties of highly textured YBa_2_Cu_3_O*_y_* ceramics was studied by Bobylev et al. [9]. It was shown that the critical current density and the first critical field, in the samples with high oxygen content (*y* = 6.96) hydrogenated at *T* = 150 °C and subsequently oxygenated, increased in comparison to the initial state. According to the authors, partial reduction of copper through the creation of Cu_2_O microinclusions and other products of chemical decomposition, which are effective pinning centers for superconducting vortices, occurs after hydrogenation.

For iron-based superconductors, it was noticed that hydrogen plays an important role both in the synthesis process and in the modification of superconducting properties, especially increasing *T*_c_ in some compounds [10]. Nakamura and Machida [11] tried to explain the origin of *T*_c_ enhancement due to hydrogen doping using first-principle calculations. They concluded that the most stable location of hydrogen atoms in 1111-type LaFeAsOH*_x_* superconductors is the neighborhood of Fe sites and this results in modification of the crystal structure that makes it more favorable for the occurrence of superconductivity.

It was shown that for niobium chalcogenides, the quasi two-dimensional crystalline structure of these compounds is very sensitive to the presence of non-magnetic atomic and molecular impurities (hydrogen, air components, etc.) [12,13].

It is well established now that the incorporation of hydrogen into iron chalcogenide superconductors (ICS), applying various methods, changes their superconducting state properties. The bombardment of FeSe with hydrogen ions leads to an increase in the sharpness of the superconducting transition [14]. The electrochemical introduction of hydrogen ions caused enhancement of superconducting transition temperature *T*_c_ from 6 to 43.5 K for FeSe_0.93_S_0.07_ and from 8.5 to 41 K for FeSe [15]. Several studies on the annealing effect of the Fe-Te-Se superconducting family, both in reactive and inert atmospheres, have been conducted up to now (e.g., [16] and references therein). However, to our best knowledge, none of the studies performed in assistance of the hydrogen atmosphere reported systematic evolution of macroscopic superconducting parameters. Annealing of the Fe-Te-Se compound in an oxygen atmosphere at temperatures of 300–400 °C leads to a strong change in superconducting properties [16] and subsequent annealing of that complex in hydrogen causes an increase in the sharpness of the transition to superconducting state [17]. Finally, the chemical combination of FeSe with tetrabutyl ammonium (TBA), each molecule of which contains 36 hydrogen atoms, shifted a critical temperature in (TBA)_0.3_FeSe to the level of 50 K (from *T*_c_ = 8 K for FeSe), which is currently record-breaking for bulk single crystals of this family of ICSs [18].

Hydrogen-assisted thermal treatment should be considered in a fourfold manner. Firstly, it has a purely thermodynamic effect, whereby annealing (micro-)structural changes can be introduced. Secondly, hydrogen can act as a reducing agent and, for example, it can affect the amount of interstitial Fe in the structure [16]. Finally, hydrogen absorption in the crystal lattice or creation of new chemical bonds can occur. Recently performed X-ray structural studies of Fe-Te-Se [19] showed that treatment in air changes the interlayer interaction in the tetragonal phase of the Fe-Te-Se and has a significant effect on the structural parameters of this superconductor. Pronounced structural changes occur under the influence of diffuse penetration of hot hydrogen (at the temperature of about 200 °C) into the sample. At this temperature, a structural phase transition of the tetragonal lattice to the orthorhombic one is observed. Moreover, the interaction of hydrogen with the matrix is changed at transition temperature, i.e., the van der Waals interaction in the tetragonal structure is changed to the chemical one in the orthorhombic phase. The latter becomes possible due to thermocatalytic dissociation [20] of molecular hydrogen in the FeTe_0.65_Se_0.35_–H_2_ system. It should be noted that the phase transition is observed only due to the impact of hydrogen atoms.

In this paper, the correlation between structural changes and superconducting properties observed for hydrogenated FeTe_0.65_Se_0.35_ single crystals was studied. The very significant increase in the critical current density after hydrogenation is explained by the appearance of additional pinning centers that were formed due to the significant mechanical stresses associated with the rearrangement of the crystal lattice after hydrogenation.

## 2. Materials and Methods

The studied crystals of FeTe_0.65_Se_0.35_, usually in the form of well-developed plates, with a tetragonal lattice of *P*4/*nmm* symmetry at room temperature and with the crystal lattice parameters: *a* = *b* = 3.799 Å, *c* = 6.093 Å, *V* = 87.9 Å^3^ [21,22], were grown from a melt by the Bridgman method. The samples were prepared from stoichiometric quantities of Fe chips (3N5), tellurium powder (4N), and high-purity Se powder (5N). All of the materials were weighed and mixed in an argon-filled glove box. Double-walled evacuated (9 × 10^−5^ Pa) and sealed quartz ampoules with starting materials were placed in a furnace with an average vertical gradient of temperature equal to ∼1.0 °C mm^−1^. The material was synthesized for 6 h at temperatures up to 700 °C. After melting at ∼860–880 °C, the temperature was held for 3 h and then was reduced down at a rate of 1 °C h^−1^, and thus, the growth velocities of the crystals were equal to ∼1 mm h^−1^.

Structural studies were carried out at room temperature with polycrystalline X-ray diffractometer using radiation of Cu-K_α_ with λ = 1.54178 Å. The beta radiation of the X-ray tube was significantly attenuated using the appropriate Ni filter. The diffraction data were collected at the natural cleavage side of the crystals, which is perpendicular to the *c*-axis in the tetragonal notation.

After growing, the crystals were maintained in the air atmosphere for several weeks. In order to remove air components captured during growth and after the process, as well as to reduce the level of internal stresses, the samples were evacuated for up to 200 h at pressures of about 0.13 Pa in a stainless steel chamber. Next, the crystals were removed from the chamber and they were mechanically cleaved along one of the (00l) planes, which were tightly packed and loosely connected by the van der Waals forces. An analysis of X-ray diffraction patterns taken from freshly cleaved crystal yielded data for a pure crystal. At the next stage, all of the studied samples were subjected to prolonged exposure to hydrogen at various temperatures for a sufficiently long time to reach hydrogen saturation. The samples were hydrogenated during 10–90 h (shorter time for higher temperatures) at the temperatures of 20, 100, 150, 180, 200, and 250 °C at a hydrogen gas pressure of 5 × 10^5^ Pa, and structural measurements were repeated.

Magnetic characteristics were studied in the temperature range from 5 to 300 K, in magnetic fields up to 70 kOe, with MPMS-7 SQUID magnetometer, and the dependences of the magnetic moment on temperature and magnetic field were recorded. Typical dimensions of the studied crystals were equal to about 2 × 2.5 mm^2^ in the *a*-*b* plane and to about 1 mm along the *c*-axis. Superconducting transition temperature was determined from zero-field cooling *M*(*T*) measurements. The critical current densities were evaluated from hysteresis loops recorded at fixed temperature, using the Bean model.

Continuous wave EPR spectra were recorded using a Bruker X−/Q− band E580 FT/CW ELEXSYS spectrometer within the temperature range of 4–18 K. For the measurements, the ER 4122 SHQE Super X High-Q cavity with TE011 mode was used. The samples were placed into quartz rods of 4 mm in diameter. The experimental parameters were: microwave frequency, 9.407 GHz; microwave power, 0.1500 mW; modulation frequency, 100 kHz; modulation amplitude, 0.2 mT; and the conversion time of 60 ms.

## 3. Results and Discussion

### 3.1. Structural Properties

The X-ray data obtained for non-hydrogenated crystal of FeTe_0.65_Se_0.35_, grown at a small speed of 1 mm h^−1^, confirmed its very high crystal perfection [22]. The X-ray patterns received from the mirror-like reflecting cleavage planes of the system can be indexed with (001) Miller indices [22]. They are the union planes of the strongly anisotropic tetragonal crystal, as has been earlier discussed in several papers [23,24,25]. The cleaving surfaces shape, and the X-ray patterns approved, the one-phase state and high perfection of the crystal grown at a small speed. They contain only tetragonal phase reflections and exhibit very high intensity and small broadening of the 004 diffraction peak [22].

**Figure 1:**
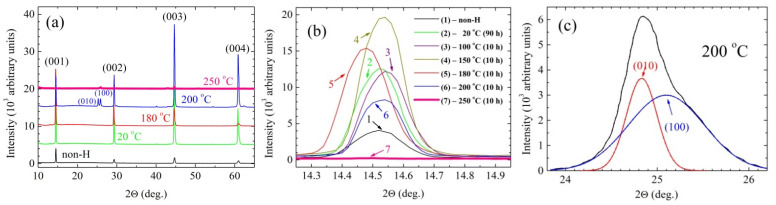
(**a**) X-ray diffraction patterns for FeTe_0.65_Se_0.35_ crystals obtained for the initial state after prolonged exposure to air and after exposing the crystal to hydrogen at a pressure of 5 × 10^5^ Pa at a temperature of 20 °C for 90 h, 180 °C for 10 h, 200 °C for 10 h, and 250 °C for 10 h. All of the diffractograms have been shifted vertically, by 5 × 10^3^ between each pattern, for clarity. (**b**) Changes in the structural characteristics of the first diffraction line (001) after the hydrogenation: (1) initial state after prolonged exposure to air, (2) after exposing the crystal to hydrogen at a pressure of 5 × 10^5^ Pa at temperature of 20 °C for 90 h, (3)−exposing at 100 °C for 10 h, (4) exposing at 150 °C for 10 h, (5) exposing at 180 °C for 10 h, (6) exposing at 200 °C for 10 h, (7) exposing at 250 °C for 10 h. (**c**) Formation of a qualitatively new diffraction pattern, displaying a rather intense asymmetric maximum at reflection angles 2Θ ~ 24–26°, appearing after exposing the crystal to hydrogen at temperature of 200 °C. Black line is a sum of red (peak 010) and blue (peak 100) lines.

Analyzing the obtained diffraction patterns, special attention was paid to the effect of hydrogen on the intensity, half-width, and structure of the studied crystals. It was found that in the studied temperature range of hydrogenation, 20–250 °C, with increasing temperature, the mechanisms of hydrogen sorption and crystal symmetry change (see, Figure 1). The tetragonal phase with the van der Waals interaction of hydrogen molecules with matrix is stable at low temperatures. Due to the catalytic effect of Fe atoms in FeTe_0.65_Se_0.35_ + H_2_ solutions at about 200 °C, hydrogen molecules (possibly also impurity oxygen molecules at lower temperatures) dissociate and the volume of the crystal unit cell decreases strongly (by 15% after hydrogenation at a temperature of 250 °C) [19]. This process is accompanied by a sharp increase in the concentration of hydrogen ions and an increase in the magnitude of stresses. Due to the large difference in the size of interacting particles in the resulting substitution solutions, an increase in the amount of local displacement defects and internal stresses may occur as a result of an increase in the concentration of hydrogen ions. At certain temperatures and external pressures, the internal chemical pressure reaches a critical value. As a result of the combination of these factors, the tetragonal lattice of iron chalcogenide loses stability, which entails a structural phase transition, at which point the relaxation of internal local stresses can occur, and the crystal symmetry decreases to orthorhombic one. From Figure 1b, it follows that the phase transition occurs near a temperature of 200 °C, where the form of diffraction patterns changes. In the temperature range of 20–180 °C, X-ray diffraction patterns of FeTe_0.65_Se_0.35_ single crystal, incubated for 10 h in hydrogen under an external pressure of gaseous hydrogen of 5 × 10^5^ Pa, contain only reflections (00l) from the basal planes of the tetragonal lattice (Figure 1a). With further heating to near 200 °C, an additional doublet of (010) (100) reflections of the orthorhombic lattice arises (Figure 1a,c). An abnormal change in the intensity and half-width of the lines of diffraction reflections also occurs in the region of structural phase transition (Figure 1b). In Figure 1b, diffraction pattern (2) shows that the intensity of the X-ray (001) reflections of the nonhydrogenated sample (diffraction pattern (1)) almost triples and its half-width decreases considerably as a result of hydrogenation for prolonged time of 90 h. A similar effect is obtained as a result of hydrogenation for 10 h at 100 °C. Exposure to molecular hydrogen H_2_ at a higher temperature of 150 °C results in a further increase in the intensity of reflections from (001) planes [pattern (4)] and, finally, in a slight decrease of its intensity for the samples hydrogenated at 180 and 200 °C (patterns (5) and (6), respectively). Finally, a slight shift toward larger angles can be observed for the sample hydrogenated at 200 °C (pattern (6)), which indicates an increase of the *c*-lattice constant. Changes observed in the diffraction pattern are most probably due to the intercalation of H_2_ molecules into the interplanar regions (the physical sorption effect). Averaged molecular interactions in such a solid solution affect atomic vibrations, which is reflected in change of relative intensities of diffraction reflections. It should be stressed that the tetragonal structure of the crystals remains. Warming of FeTe_0.65_Se_0.35_ crystals, even to the relatively low temperature of *T* ≥ 200 °C, in a hydrogen atmosphere at a pressure of 5 atm leads to the formation of a qualitatively new diffraction pattern. The X-ray diffraction patterns reveal a rather intense asymmetric maximum (Figure 1c) at reflection angles 2θ ~ 24–26°. Simultaneously, the intensity and angular location of the (001) reflections change slightly. One may assume that the observed change in the diffraction pattern is due to the structural phase transition from tetragonal to orthorhombic phases. The transition is started by thermocatalytic dissociation of H_2_ molecules and by filling of interatomic vacancies in the basal planes by atomic hydrogen. According to the data provided in Ref. [20], such a process is quite possible in the investigated system. The dissociation of hydrogen molecules takes place at the catalytically active centers of metals from the iron group at *T* > 200 °C [20]. The existence of atomic hydrogen in the basal planes can considerably influence interatomic interaction, strengthen its anisotropy, and, as we noticed, lower the symmetry of the crystal structure from the tetragonal to orthorhombic phase. As a consequence of reducing the perfection of FeTe_0.65_Se_0.35_ crystals, one can observe the (010) and (100) reflections of the orthorhombic structure (Figure 1c), together with the (001) reflections. Additional small reflections observed for some crystals after hydrogen post-annealing are not fully understood but can be linked with some site phases, such as Fe-O ([16,26] and references therein) or Fe_7_Se(Te)_8_ [24]. However, the effect can be also linked with microstructural changes caused by thermal treatment in the hydrogen atmosphere, which cause the appearance of lower index reflections.

### 3.2. Superconducting Properties

**Figure 2:**
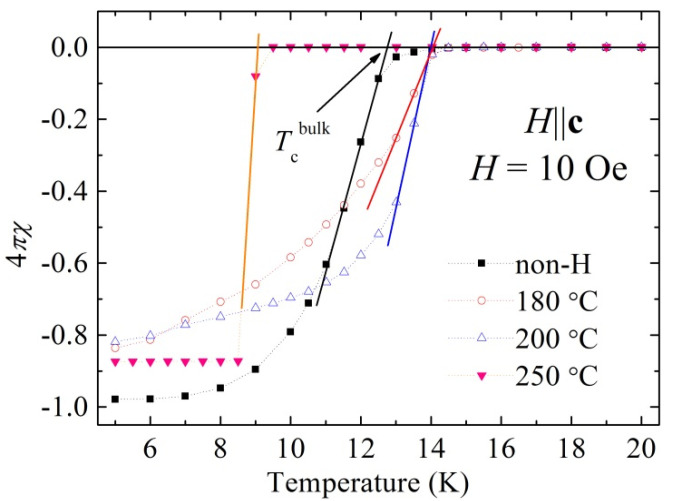
Temperature dependence of dc magnetic susceptibility, recorded in zero field cooling mode in *H* = 10 Oe parallel to the *c*-axis, for the as-grown single crystal of FeTe_0.65_Se_0.35_ and after hydrogenation at 180, 200, and 250 °C.

Figure 2 shows temperature dependence of dc magnetic susceptibility χ(*T*) of the studied crystals in the region of the superconducting phase transition. The χ(*T*) curves recorded in ZFC measurements performed for *H* parallel to the *c*-axis in external magnetic field of 10 Oe are shown for both the pristine sample and after hydrogenation at temperatures of 180, 200, and 250 °C for 10 h. All data were corrected for demagnetizing field. The onset of transition to superconducting state, *T*_c_^onset^, for the pristine sample and for the samples hydrogenated at temperatures of 180 and 200 °C is almost identical. Diamagnetic signal appears at about 15.5 K, which is in perfect agreement with the superconducting onset temperature data for pristine sample published by Sivakov et al. [22], while for the sample annealed at 250 °C, the value of *T*_c_^onset^ is significantly reduced and is equal to about 9.5 K, which may indicate a partial degradation of the sample annealed at a temperature above 200 °C. However, there is substantial difference in the value of bulk critical temperature, *T*_c_^bulk^. This temperature is defined by a intersection of linear extension of normal state susceptibility to low temperature with linear extension of diamagnetic susceptibility to high temperatures from the temperature range far below the transition (see, schematic plot defining *T*_c_^bulk^ given in Figure 2). One can see that the *T*_c_^bulk^ increases from 12.75 K for the non-hydrogenated single crystal (the value being very similar to that of non-hydrogenated crystal of FeTe_0.65_Se_0.35_, grown at a small speed of 1 mm h^−1^ [22]) to 14.05 and 14.0 K for the samples hydrogenated at temperatures of 180 and 200 °C, respectively. Hence, the *T*_c_^bulk^ increases by more than 1 K as a result of hydrogenation at 180 and 200 °C. There is an insignificant decrease in diamagnetic response at 5 K as a result of hydrogenation at 180 and 200 °C, but despite that, it is suspected that hydrogen annealing affects the magnetic response only slightly. However, H_2_ could partly reduce already formed impurities of Fe_3_O_4_ or Fe_2_O_3_ to ferromagnetic Fe and H_2_O. Hence, the magnetic component might change from iron oxide to Fe upon H_2_ annealing, resulting in different magnetic moments of iron oxide and Fe, respectively. It may reduce, to some extent, the diamagnetic volume fraction, and thus reduce the diamagnetic response of the sample. Magnetic impurities may destroy local superconductivity—they can lead to a local decrease in superconducting carrier density and an increase in penetration depth, and thus a reduction of diamagnetic response. We already noticed small decreases of superconducting responses in superconducting FeTe_0.5_Se_0.5_, FeTe_0.66_Se_0.34_, and Fe_0.994_Ni_0.007_Te_0.66_Se_0.34_ when a degradation of crystal quality under applied elevated pressure was correlated with significant improvement of superconducting state properties [27]. After treatment with hydrogen at temperature of 250 °C, the *T*_c_^bulk^ decreases to about 9 K. Importantly, the *M*-*H* curve for the sample hydrogenated at 250 °C is completely different from that for pristine sample and for the samples hydrogenated at 180 and 200 °C. Extremely sharp diamagnetic response—evidenced by the change from normal state susceptibility at 9.05 K to the constant, temperature-independent, bulk superconductivity value below 8.5 K—is observed. It may indicate that a new superconducting phase with transition temperature in the range of about 8.5–9.1 K is formed following to complete degradation of the 14–15 K superconducting phase as a result of hydrogenation at 250 °C. Superconducting properties of the phase with *T*_c_ in the range of about 8.5–9.1 K will be a subject of separate studies [28]. Comparison of the structural data (Figure 1) and observed tendency in the critical temperature shows that an increase in *T*_c_^bulk^ observed after hydrogenation at 180 and 200 °C apparently correlates with an increase in the intensity, width, and angular position of the (001) diffraction peak.

An essential property of a superconductor, which determines its magnetic and current-carrying abilities, is the capability to pin vortices trapped in the volume. To study the dynamics of magnetic flux in FeTe_0.65_Se_0.35_, the measurements were carried out in the field cooling mode (FC). When the magnetic field is turned off, the residual magnetization of the sample and its dynamic are determined by the state of the captured magnetic flux in the bulk of the superconductor. As the thermally activated creep of individual vortices and their bundles can lead to the redistribution and weakening of bulk superconducting currents, the integral magnetic moment *m*(*t*) in the superconductor may decrease with increasing time and the averaged magnetization *M* of the superconducting sample can relax in time [22]. The role of near-surface energy barriers (for example, the Bean–Levingston barrier [29]), which are difficult to control and analyze, in the dynamic of magnetic flux under these conditions is minimal. Importantly, we found that relaxation for the samples hydrogenated at temperatures of 180–250 °C was much smaller than that for the non-hydrogenated one; in fact, it was practically absent. This indicates that hydrogenation at temperatures as low as 180–200 °C forms new and very powerful vortex pinning centers of the crystals, and their concentrations, rather than their pinning potential, changes with variation of hydrogenation conditions.

In the case of the FeTe_0.65_Se_0.35_ crystal, one should take into account the presence of excess Fe, as well as magnetic inclusions, such as Fe_3_O_4_ and Fe_7_Se_8_, which are located inside the superconductor and on its surface [22,23,24]. As a result, the magnetization of sample *M* is expected to be a superposition of two contributions *M* ≈ *M*_F_ + *M*_D_, where *M*_F_ and *M*_D_ are the ferromagnetic and diamagnetic contributions, respectively. As a result, the magnetization loop *M*(*H*) may become asymmetric with respect to the *H*-axis [21,22,30]. However, the asymmetry of the hysteresis loop may be caused by the influence of the Bean–Levingston surface barrier too [29].

**Figure 3:**
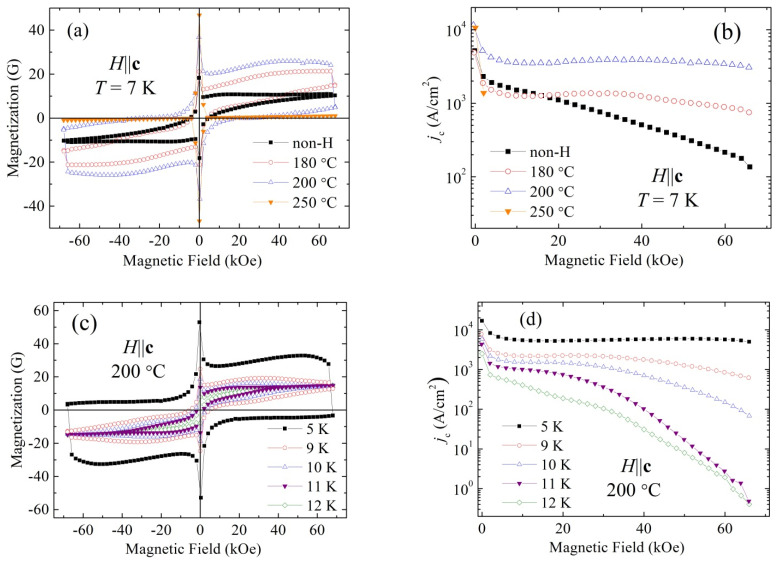
(**a**) Magnetization hysteresis loops recorded at 7 K for pristine FeTe_0.65_Se_0.35_ crystal and for the crystals hydrogenated at 180, 200, and 250 °C. (**b**) Field dependence of the critical current density, *j*_c_, at 7 K for the FeTe_0.65_Se_0.35_ pristine single crystal hydrogenated at 180, 200, and 250 °C. (**c**) Hysteresis loops recorded in the temperature range from 5 to 12 K for the crystal hydrogenated at 200 °C. (**d**) Comparison of field dependence of *j*_c_, recorded at various temperatures in the temperature range from 5 to 12 K, for the crystal hydrogenated at 200 °C.

The impact of an uncompensated magnetic moment of Fe ions and/or other Fe-based inclusions on the shape of hysteresis loop in the studied materials seems to be dominant. The asymmetry of the loop for the sample hydrogenated at 250 °C is significantly reduced compared to that for the as-grown sample and for the samples hydrogenated at 180 and 200 °C. However, the shape of reversible contribution to hysteresis loop, recorded in quite a wide temperature range from 5 to 12 K for the sample hydrogenated at 200 °C (see Figure 3c), remains practically unchanged despite significant changes in superconducting state properties. This indicates that this contribution is of magnetic origin. Importantly, we did not observe difference between magnetization recorded with increasing and decreasing magnetic field for the temperatures just above the superconducting state transition. This means that uncompensated magnetic moments of Fe ions and/or other Fe-based inclusions influence the shape of the hysteresis loop in the studied materials, but do not influence the critical current density evaluated based on the Bean critical state model [31,32].

According to the Bean critical state model, the critical current density *j*_c_ can be estimated using the well-known formula *j*_c_ = 20Δ*M*/[*a*(1−*a*/3*b*)], where *a*, *b* (*a* < *b*) are the sizes of the cross-section of the sample and Δ*M* is the width of the *M*(*H*) loop [33]. As one can see in Figure 3a, the width of the hysteresis loop, Δ*M*, is significantly larger for hydrogenated crystal as compared with the pristine one, and only slightly decreases with increasing field up to 70 kOe. Apparently, the defects generated in the structure during hydrogenation are very effective pinning centers. The largest Δ*M* is observed for the crystal hydrogenated at 200 °C. This correlates well with the observed structural rearrangement observed for FeTe_0.65_Se_0.35_ crystal due to hydrogenation. As it was shown above, at 200 °C, the structural phase transition of the tetragonal lattice to the orthorhombic one is observed. On the other hand, the hydrogenation at 250 °C leads to partial amorphization of FeTe_0.65_Se_0.35_ compound [19]. In consequence, the superconducting properties of critical temperature and capability to carry the superconducting current are much poorer. It can be assumed that its diamagnetic properties will be degraded. Measurements of the *M*(*H*) dependence for the sample processed at temperature of 250 °C completely confirmed this assumption. The diamagnetic magnetization of the crystal significantly decreases even in relatively weak fields (less than 100 Oe), and at large fields it disappears completely (Figure 3a).

Figure 3b shows the values of the critical current density of the crystals before and after their treatment with hydrogen, based on the *M*(*H*) dependences presented in Figure 3a. The width of the hysteresis loop for a single crystal before hydrogenation, presented in Figure 3a, for the same temperature is not greater than that for the crystal hydrogenated at 180 °C. However, the *j*_c_ values for pristine crystal at low fields are slightly larger than those for the crystal hydrogenated at 180 °C because of some differences in the size of studied crystals, affecting the width of measured hysteresis loop. It can be apparently seen that treatment with hydrogen increases the value of *j*_c_. The highest values of *j*_c_ are observed for the crystal hydrogenated at 200 °C. Particularly spectacular is the increase of *j*_c_ at higher magnetic fields. In fact, for fields higher than 5 kOe, the *j*_c_(*H*) dependence at lower temperatures is almost flat, both for the crystals hydrogenated at 180 and 200 °C. For the crystal hydrogenated at 200 °C, at high magnetic fields, *j*_c_ is at a level of 3 × 10^3^ A/cm^2^). This shows that introduced pinning centers are very robust and effective in high magnetic fields.

In the crystal hydrogenated at 200 °C, the critical current density at a temperature of 7 K, in fields up to 20 kOe, is about 4 times higher than for pristine crystal. This difference increases in the fields higher than 20 kOe, and in the field of 70 kOe, the ratio of both critical current densities reaches the value of about 30 (see, Figure 3b). It should also be noted that in the range of fields of 10–70 kOe, *j*_c_ of such a crystal practically does not change at low temperatures. The maximum value of *j*_c_ in the zero field at a temperature of 7 K is 10^4^ A/cm^2^. Somewhat unexpected is the same high critical current density in a zero field in a crystal treated at a hydrogen temperature of 250 °C, although the superconductivity in it is destroyed even in a weak magnetic field.

Evolution of *j*_c_(*H*) with temperature for the sample hydrogenated at 200 °C is presented in Figure 3d. Obtained data show high *j*_c_ values for the fields below 60 kOe and temperatures below 9 K. A significant increase in the critical current density caused by saturation of the sample with hydrogen at 200 °C is not surprising. It was already demonstrated that oxygen treatment of Fe_1+*x*_Te_1-*y*_Se*_y_* at 300 °C improves its superconducting properties through irreversible oxidative de-intercalation of interstitial iron atoms. In such a process, traces of magnetic iron oxides are formed. The heterogeneous reaction begins at the surface and probably causes inhomogeneous particle distribution, accompanied by FeTe_2_ impurity formation. Thus, the anti-PbO-type phase obviously degrades if iron is extracted from the layers of FeTe_1-*y*_Se*_y_* tetrahedra [16,17].

As the volume of the crystal lattice decreases by about 15% after hydrogenation [19], this leads to large chemical pressure effect. Hence, the observed increase in the critical current density can be explained by the appearance of additional pinning centers due to significant mechanical stresses associated with the rearrangement of the crystal lattice after hydrogenation.

### 3.3. EPR Studies

Given that the most pronounced improvement of superconducting properties was observed for the crystal hydrogenated at 200 °C, the EPR studies were performed for the pristine (not hydrogenated) crystal and for the crystal hydrogenated at 200 °C.

**Figure 4:**
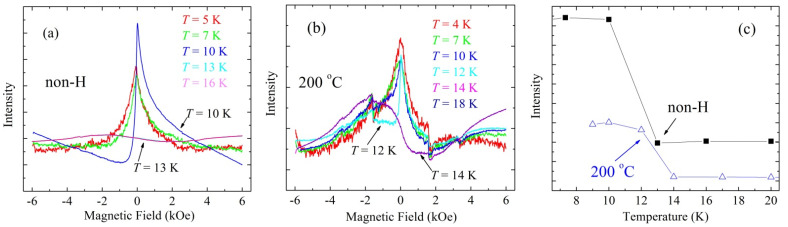
(**a**,**b**) Wide asymmetric lines observed in the temperature range of 4–18 K, which change significantly with decreasing temperature from 13 to 10 K (**a**), and from 14 to 12 K for the crystal hydrogenated at 200 °C (**b**). (**c**) Temperature dependence of the integrated intensity of the absorption line in the region close to the bulk superconducting transition at about 13 K for both the as-grown and hydrogenated at 200 °C single crystals of FeTe_0.65_Se_0.35_.

An absorption line in the zero field is clearly seen for both studied crystals. However, a substantial difference between the samples is observed in the form of an EPR line at the superconducting transitions. For the as-grown crystal, a significant change in the absorption line occurs in the temperature range *T* = 10–13 K, which corresponds to superconducting phase transition in the material (see, Figure 4a). For the crystal hydrogenated at 200 °C, a wide asymmetric line is observed in the temperature range of 4–18 K, which changes significantly with decreasing temperature from 14 to 12 K, which can be characterized as a result of the phase transition (see, Figure 4b). This means that the transition to a superconducting state for the sample hydrogenated at 200 °C is located in a higher temperature range than that for the as-grown sample. At temperatures above *T*_c_, the dependence of the EPR line on temperature was not observed. Temperature dependence of the integrated intensity of the absorption line in the region close to the bulk superconducting transition at about 13 K for both the as-grown and hydrogenated at 200 °C single crystals is presented in Figure 4c.

## 4. Conclusions

It was shown that hydrogenation performed at temperature 180–200 °C strongly improves superconducting properties—bulk critical temperature increases by more than 1 K and transition to superconducting state becomes sharper. Additionally, the new, very effective pinning centers are introduced, which results in a strong increase in the value of the critical current density (4–30 times) compared with the pristine sample. After hydrogenation, relaxation of magnetization is practically absent. The hydrogenation causes pronounced changes in the crystallographic structure and enhances the superconductivity in this system. Conclusions that result from the magnetic measurements are in line with those drawn from the transport measurements performed already on iron-based chalcogenide crystals of various crystallographic quality [22]. However, the improvement of superconducting properties at ambient pressure, reported here, was obtained as a result of degeneration of crystallographic quality in the hydrogenation process for a specific crystal. Importantly, it was not a result of tailoring crystal growth conditions, leading to the growth of crystals with distinct crystallographic qualities, and thus with distinct superconducting properties. EPR studies confirmed the shift of bulk superconducting transition to a higher temperature range as a result of hydrogenation.

## Data Availability

The data that support the findings of this study are available from the corresponding author upon reasonable request.
